# Identification of a cantilever beam’s spatially uncertain stiffness

**DOI:** 10.1038/s41598-023-27755-5

**Published:** 2023-01-20

**Authors:** Karl-Alexander Hoppe, Martin G. T. Kronthaler, Kian Sepahvand, Steffen Marburg

**Affiliations:** TUM School of Engineering and Design, Department of Engineering Physics and Computation, Chair of Vibroacoustics of Vehicles and Machines, Technical University of Munich, 85748 Garching bei München, Germany

**Keywords:** Mechanical engineering, Mechanical properties, Characterization and analytical techniques

## Abstract

This study identifies non-homogeneous stiffnesses in a non-destructive manner from simulated noisy measurements of a structural response. The finite element method serves as a discretization for the respective cantilever beam example problems: static loading and modal analysis. Karhunen–Loève expansions represent the stiffness random fields. We solve the inverse problems using Bayesian inference on the Karhunen–Loève coefficients, hereby introducing a novel resonance frequency method. The flexible descriptions of both the structural stiffness uncertainty and the measurement noise characteristics allow for straightforward adoption to measurement setups and a range of non-homogeneous materials. Evaluating the inversion performance for varying stiffness covariance functions shows that the static analysis procedure outperforms the modal analysis procedure in a mean sense. However, the solution quality depends on the position within the beam for the static analysis approach, while the confidence interval height remains constant along the beam for the modal analysis. An investigation of the effect of the signal-to-noise ratio reveals that the static loading procedure yields lower errors than the dynamic procedure for the chosen configuration with ideal boundary conditions.

## Introduction

Material parameters may be identified in various ways. The established methods can be categorized as destructive and non-destructive methods^1^. “Destructive” implies that the measurement specimen has, for example, experienced plastic deformations during tensile tests and thus fails to comply with the product requirements after the test, i.e. it can no longer fulfill the original purpose. Often, these tests are carried out until the specimen fails. Non-destructive testing methods offer a way to identify material parameters while the specimen retains its properties. Therefore, these methods are popular for quality control purposes after the manufacturing process in order to ensure certain requirements.

On the one hand, dynamic methods are popular for testing engineering materials. Impact-echo or transmission measurements using elastic waves present popular high-frequency regime methods that evaluate the wave onset^2^. However, considering the individual modes of guided ultrasonic waves contains more information^3–5^. In general, wave fitting approaches in the high-frequency regime continue to evolve^6^, where the utilization of the full waveform is noteworthy^7^. In lower frequency regimes, standing waves can be utilized. In this case, the resonance frequency method uses the eigenfrequencies connected to the eigenmodes for material parameter identification or defect detection^8^.

On the other hand, static methods may be considered as non-destructive when they are reversible and place the specimen in linear elastic loading conditions. Indentation tests and strain measurements with strain gauges are used in procedures that operate at the surface level, just as many displacement measurement techniques do. Within the latter, digital image correlation between a reference state and the deformed state of a specimen leads to a displacement field^9^, where several techniques can be used for capturing the respective images^10^.

Discontinuities like defects or cracks are typically the quantities of interest for nominally homogeneous materials^11^. With non-homogeneous materials, local spatial variation of material properties is additionally introduced into the system^12^. Depending on the severity of the non-homogeneity, it may have a relevant effect on the system response. This is certainly the case for engineering materials such as wood. The spatial variation of material properties has been quantified for individual specimens^13,14^. Savvas et al.^15^ identify the mesoscale spatial variation of material properties given microscale information. However, rigorous descriptions of the spatial behavior are not readily available. Given this lack of data, the standard procedure is to assume a random spatial variation of the material properties. This spatial randomness of material properties can be described with the theory of random fields, which is extensively treated in the literature^16,17^. Rasmussen and Williams^18^ popularize this theory for regression, which is generalized by Duvenaud^19^. The integration of spatial uncertainties with the finite element method (FEM) is covered in the literature^20,21^.

Spatial uncertainty is thus compatible with established uncertainty quantification practices^22^. Sepahvand and Marburg^23^ demonstrate this for the forward propagation of uncertainty in structural dynamics by representing material properties as random fields.

Knowledge of the sensitivities of the system outputs with respect to the system inputs is valuable. However, many non-destructive testing methods involve an inverse problem, as for instance the study on elasticity imaging by Gokhale et al.^24^. Since the quantities of interest as well as the measured parameters are fraught with uncertainties, a natural approach for the solution of the aforementioned inverse problems lies in Bayesian inference^25–27^.

Parameter identification using the Bayesian framework holds two major advantages over other methods. Firstly, when limited test data on parameters exist, Bayesian methods provide us with an optimal tool to quantify uncertainty^28^. This is crucial when one deals with expensive experiments in engineering. Using classical frequentist statistical models for such situations only yields reliable results when the number of data points is larger than a specific number, mostly 30, or when the data strictly follows a normal distribution^29^. If these criteria are not met, the results generated with these methods either cannot be trusted to be valid or involve an increased level of uncertainty.

Secondly, the Bayesian framework involves available prior information about parameters that the statistical model considers^30^. This prior information is then updated by information gained from observations. Available sources of prior information may include primary data, literature, online databases, and even the knowledge of experts. This is a substantial argument for using Bayesian methods in engineering applications, where data may be scarce but expertise on parameters is abundant.

Marzouk and Najm^31^ pioneer the application of Bayesian inference to spatially varying quantities of interest via dimensionality reduction achieved by the Karhunen-Loève (KL) expansion. They use a surrogate for the forward model to reduce computational cost that is based on generalized Polynomial Chaos (gPC)^21^. The decoupling of spatial discretization of the computational domain from the random dimensionality makes inverse problems involving larger systems accessible.

Sun and You^32^ provide an overview of sensitivities and damage features related to modal analysis in the context of non-destructive testing. Cugnoni et al.^33^ perform a deterministic identification of a composite plate material model using the combined information of natural frequencies and mode shapes. Sepahvand and Marburg^34,35^ compute the homogeneous elastic parameters of composite plates while accounting for uncertainty using experimental data. Note the contribution by Desceliers et al.^36^, who calculate the non-homogeneous beam stiffness from frequency response measurements using a maximum likelihood estimate. Batou and Soize^37^ consider a random field material model employing model order reduction and maximum likelihood estimation given frequency response functions. Mehrez et al.^38^ estimate the Young’s modulus of a composite structure at a set of nodes with Bayesian inference and gPC using frequency response functions acquired at those nodes. Debruyne et al.^39^ apply this general procedure to a honeycomb structure.

This study investigates the identification of spatially varying structural flexibility using both a dynamic and a static method. The dynamic method is a novel dimensionality-reduced Bayesian approach for identifying the elastic parameters of a structure using resonance frequency information. The static method follows a similar scheme as the research by Uribe et al.^40^, who reconstruct the stiffness fields given deflection observations using a modified version of the framework by Marzouk and Najm^31^.

To provide comparability and insight into each method’s respective advantages, both the dynamic and static method use the same setup, namely a cantilever beam with spatially varying structural flexibility. Eigenfrequencies mark the starting point for the flexibility identification within the dynamic method, while deflections connected to static loading serve as data for the static method. For each method, Bayesian updating is then performed on a finite element method model of the cantilever beam with unknown structural flexibility, which is considered as a sample of a Gaussian random field along the cantilever beam. The truncated KL expansion represents this spatially varying flexibility, resulting in a description with reduced random dimensionality. Owing to the Bayesian inference setup, the solution’s uncertainty can then be compared between the dynamic and the static approach.

This paper is organized as follows: “Methods” introduces random fields and inverse problems, as well as the Bayesian inference setup shared between the dynamic and static approaches. “Application of the procedure” describes the integration of both the dynamic and static cantilever beam models into the inverse problem, and then the numeric results are presented in “Results and discussion”. Following the conclusion and an outlook on future research in “Conclusion”, we provide additional information in the Online appendix S1.

## Methods

This study considers the spatially random fluctuation of material properties about a mean value. The connected covariance and the representation by the KL expansion are covered by “Preliminary concepts” alongside Bayes’ theorem. “Procedure” treats the inverse problem formulation and the latter’s integration into Bayesian updating by specifying the parametrization and measurement error model pertinent to the cantilever beam.

### Preliminary concepts

Together with its mean value, a second-order random field is fully characterized by its covariance function. The covariance kernel $Cov(t, t')$ is a function of the coordinates of two points $t, t'$ within the field’s domain, the bounded interval [0, *L*]. This study considers continuous, symmetric, and positive semi-definite kernels such that the KL expansion can be used.

Several families of functions may be used as covariance functions. We adopt the isotropic exponential kernel from the literature^17^. It is a function of Euclidean distance *r* and the length scale parameter *l* as

1 $$\begin{aligned} Cov(t, t') = \sigma ^2\exp \left( -(\vert t - t'\vert /l)^2\right) , \end{aligned}$$

where $\sigma ^2$ is the variance^18^. It is chosen because there exist analytical solutions to the connected eigenvalue problem that facilitate verifying the corresponding numerical implementations^41^.

#### Karhunen-Loève Expansion

The KL expansion represents a random field by taking into account the random field’s mean $\mu (t)$ and decomposing its covariance function. This method utilizes deterministic spatial functions together with random coefficients $\xi _i$ for the representation of the random field. Truncating the KL expansion after *s* summands yields an approximation of the field with a finite random space dimensionality^42^, such that

2 $$\begin{aligned} X(t, \xi ) \approx \mu (t) + \sum _{i=1}^s \sqrt{\lambda _i}\;\varphi _i(t)\;\xi _i{{,}} \end{aligned}$$

where $\lambda _i$ are the eigenvalues and $\varphi _i(t)$ are the eigenfunctions of the corresponding covariance operator^42^. To obtain a sample path or realization of the random field, a sample of its parametrization $\varvec{\xi }$ must be drawn.

If the considered material parameter follows a lognormal instead of a normal distribution, the generated samples may simply be exponentiated. However, the generalization of the KL expansion to non-Gaussian random fields is not straight-forward. Partially, this is due to correlations being induced between the random coefficients. When closed-form transformations are not readily available, a full-dimensional multivariate normal distribution may present a remedy. After transformation to [0, 1] using the Gaussian error function, the inverse cumulative distribution function of a desired arbitrary distribution can be applied. The resulting marginal distributions follow the prescribed distributions and retain the sample smoothness over the domain inherent to the initial correlation structure, see Vořechovský^43^.

#### Bayesian Inference

The above describes the quantity of interest, which is now declared as $\varvec{\theta }$. The following introduces Bayesian inference, a method for estimating the quantity of interest using a model, data, and prior knowledge. Bayesian inference approaches attempt to solve the inverse problem while considering uncertainties along with prior knowledge about the quantities of interest and the likelihood of the observed data. Essentially, its outcome, the posterior, reflects how new data change our beliefs concerning the unknown quantities.

Using the logarithms of the probabilities to circumvent computational issues arising from the multiplication of small numbers and neglecting the normalizing constant that is the evidence, Bayes’ theorem reads as

3 $$\begin{aligned} q(\varvec{\theta }\vert \varvec{d}) \equiv l(\varvec{d}\vert \varvec{\theta }) + p(\varvec{\theta }). \end{aligned}$$

Here, *q* is the posterior distribution for $\varvec{\theta }$ given some data $\varvec{d}$, *l* is the likelihood of observing the data $\varvec{d}$ given a model with parametrization $\varvec{\theta }$, and lastly, *p* is the prior distribution on $\varvec{\theta }$.

The reader is referred to the literature concerning the treatment of three major issues within the solutions of inverse problems: existence, non-uniqueness, and instability of the solution, with the latter also called ill-posedness^44^.

### Procedure

**Figure 1:**
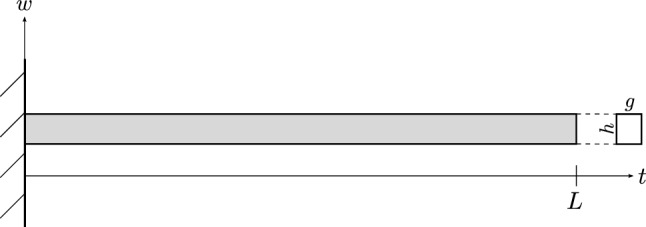
The figure shows a side view of the investigated cantilever beam model together with its profile and the coordinate system. The rectangular profile exhibits width *g* and height *h*. The beam length is *L*. Here, the beam coordinate is denoted as *t*, and the deflection coordinate reads as *w*.

Consider a forward model, see Fig. 1, of a cantilever beam

4 $$\begin{aligned} \varvec{d}_{true} = \mathscr {G}(C(t)). \end{aligned}$$

Here, its structural flexibility *C*(*t*) is considered as a function over the beam domain [0, *L*]. The operator $\mathscr {G}$ is used to transform this function to an output $\varvec{d}$. Static deflections and eigenfrequencies comprise $\varvec{d}$ for the static analysis and the modal analysis, respectively. The measured output

5 $$\begin{aligned} \varvec{d}_{meas} = \varvec{d}_{true} + \varvec{\eta } = \mathscr {G}(C(t)) + \varvec{\eta } \end{aligned}$$

is subject to measurement noise $\varvec{\eta }$. Solving the inverse problem is then to

6 $$\begin{aligned} \text {find}\quad C(t)\quad \text {s.t.}\quad \varvec{d}_{true} = \mathscr {G}(C(t)),\quad \text {given}\quad \varvec{d}_{meas}. \end{aligned}$$

In practice, a finite-dimensional representation of the flexibility *C*(*t*) based on the parameter vector $\varvec{\theta }$ made up of the KL parameters and the mean of the flexibility field reads as

7 $$\begin{aligned} {{ \varvec{\theta } = \left\{ \mu _C, \xi _{1},\xi _{2}, \dots , \xi _{s}\right\} ^T \in \mathbb {R}^{s+1}.}} \end{aligned}$$

This leads to the discretized numerical forward model

8 $$\begin{aligned} \varvec{d}_{meas} \approx \mathscr {G}(C(t, \varvec{\theta })) + \varvec{\eta } = \widetilde{\mathscr {G}}(\varvec{\theta }) + \varvec{\eta }. \end{aligned}$$

Now, Eq. (3) may be adopted to the problem at hand with $\varvec{d}=\varvec{d}_{meas}$, and the finite-dimensional parametrization $\varvec{\theta }$ given in Eq. (7). The necessary truncation order of the KL expansion depends on the covariance and is independent of the spatial discretization chosen within the forward model. To determine *s*, the ratio of the variance covered by the truncated KL expansion to that covered by the full expansion should be compared to recommended threshhold ratios^45^. Typically, *s* is less than 20, and is significantly smaller than the spatial discretization of the governing equations. This reduction in dimensionality from the spatial discretization to the number of KL coefficients is crucial for the efficiency of some Markov Chain Monte Carlo (MCMC) algorithms. Additionally, it allows for the use of surrogate model methods like gPC^31^.

Specifying the measurement noise model, a custom likelihood accommodates for flexible signal-to-noise ratios of the data components. This measurement error model assumes that the measurement vector $\varvec{d}_{meas}$ of dimension $\kappa$ is perturbed by independent noise components

9 $$\begin{aligned} \eta _j \sim \mathscr {N}(0,\ \sigma _j^2) \end{aligned}$$

with corresponding variances $\sigma ^2_j$. Now, for scalar-valued measurements at several frequencies or locations within the specimen and a single measurement run, the likelihood

10 $$\begin{aligned} \mathscr {L}(\varvec{d}_{meas}\vert \varvec{\theta }) = \prod _{j=1}^{\kappa }\frac{1}{\sigma _j \sqrt{2\pi }} \exp \left( -\frac{1}{2}\frac{(d_{meas,j}-\widetilde{\mathscr {G}}(\varvec{\theta })_j)^2}{\sigma _j^2}\right) \end{aligned}$$

becomes the product of the marginal likelihoods of its components. Vector-valued measurements as well as repeated measurements necessitate modifications of Eq. (10).

With fixed choices for the likelihood, the forward model, its parametrization and the latter’s endowment with prior densities, the right hand side of Eq. (3) can be evaluated. However, closed form solutions for the posterior probability density function are only available for special cases involving conjugacy. This necessitates sampling from the posterior, which can be achieved using Markov Chain Monte Carlo (MCMC) algorithms. This study employs the single variable slice sampling method as formulated by Neal^46^. It is applied to each parameter separately, while the other parameters are fixed.

## Application of the procedure

This section describes the application of the methods presented in “Methods”. Specifically, “Cantilever beam model” introduces the used cantilever beam model, while “Modal analysis” describes the system’s modal analysis and “﻿Static analysis” covers the system’s static analysis. After the explanations concerning these forward models, “Flexibility identification using eigenfrequency measurements from modal analysis” provides the solution procedure for the inverse problem based on modal data and “Flexibility identification using deflection measurements from static analysis” details the procedure when deflection data is given.

### Cantilever beam model

Consider the Timoshenko cantilever beam model shown in Fig. 1, where the boundaries are clamped on the left side and free on the right side. The beam exhibits length *L* and a rectangular cross-section with an area of $A = g\cdot h$, where the cross-section width and height are denoted by *g* and *h*, respectively. The second moment of area is computed as $I = g h^3/12$, and the shear correction factor $k_s$ for a rectangular cross-section is $k_s = 5/6$. The material of the beam is characterized by Young’s modulus *E* and the shear modulus *G*, while considering Hooke’s law.

This problem is implemented with the finite element method via the SfePy Python library^47^. The discretization of the deflection *w*, the angle $\psi$, and the corresponding weighting functions is performed using $2^\mathrm {{nd}}$ order polynomials that are defined on each element.

**Figure 2:**
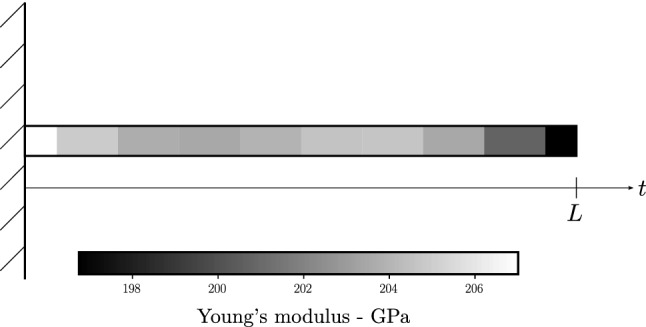
The graph shows an arbitrarily chosen stiffness distribution over the beam coordinate at ten discrete positions within the numerical model of the cantilever beam. The discretization is purposefully chosen as coarse for the illustration. Because the stiffness is assigned to nodes as opposed to elements, the stiffnesses at the bounds are half as wide compared with those assigned to interior elements.

To model the spatially varying elastic modulus *E*, it is assumed to vary randomly over the beam coordinate *t*. The inverse of the elastic modulus, i.e. the elastic flexibility $C = 1/E$, is then assumed to be a realization of a Gaussian random field, where the standard deviation is a fraction of the mean value. The covariance function for the random flexibility is defined on the domain $t\in [0,L]$ and an exponential kernel with arbitrarily chosen correlation length $l=L/5$, as defined in Eq. (1), is chosen. The covariance function is evaluated at the nodes of the finite element mesh, yielding piece-wise constant material properties as shown for a coarse exemplary discretization in Fig. 2.

The domain is discretized with 100 finite elements. This results in 201 nodes for the evaluation of the covariance function. The resulting $201\times 201$ covariance matrix is used for synthesizing the reference flexibility vector. The Cholesky decomposition $\varvec{LL}^\text{T}$ of this covariance matrix achieves the realization of the reference flexibility^20^. This alternative method is chosen for the reference model instead of the KL expansion to mitigate an inverse crime, as it is more accurate, albeit higher dimensional, than the KL expansion. With the prescribed mean bending flexibility $\mu _{C, true}$ and the lower triangular matrix $\varvec{L}$ resulting from the Cholesky decomposition, the flexibility field reads as

11 $$\begin{aligned} \varvec{C}_{true} = \varvec{\mu }_{C, true} + \varvec{L}\varvec{\xi }, \end{aligned}$$

where $\varvec{\xi }$ is a vector of uncorrelated standard Gaussian random numbers. Realizing $\varvec{\xi }$ yields the reference sample of the flexibility.

#### Modal analysis

On the one hand, we consider the modal analysis of the cantilever beam described in “Cantilever beam model”. Here, the first $\kappa$ eigenfrequencies of the system $f_1, f_2, \dots ,f_{\kappa }$ obtained via solving the system’s eigenvalue problem make up the response vector. Specifically, the reference flexibility $\varvec{C}_{true}$ of the cantilever beam leads to the connected reference eigenfrequencies. A vector of independent Gaussian random variables is then superimposed on these eigenfrequencies to emulate measurement noise.

#### Static analysis

On the other hand, we consider the cantilever beam described in “Cantilever beam model” when subjected to static loading *F* at $t=L$. Here, $\kappa$ equispaced static deflection measurements comprise the response vector. After applying the reference flexibility $\varvec{C}_{true}$ to the cantilever beam model, we calculate the connected reference deflections. To simulate measurement noise, the static deflections are superimposed with independent and identically distributed Gaussian random variables.

### Identification of spatially varying flexibility using synthetic noisy measurements

#### Flexibility identification using eigenfrequency measurements from modal analysis

Next, we use noisy measurements of the first 10 simulated eigenfrequencies of the cantilever beam with the reference flexibility vector. Then, the reference flexibility is estimated for all positions within the beam from these noisy eigenfrequency measurements. Note that the reference flexibility is unknown in the context of the inversion procedure.

Figure 4 shows a flowchart of the inference procedure, while the following paragraphs describe it in greater detail.

Reconstructing the unknown reference flexibility with the methods described in “Methods” necessitates the strong assumption of the flexibility mean being constant, that is stationary, and that of the flexibility covariance. We assume the same covariance, an exponential covariance kernel with correlation length $l=L/5$ and an exponent of $\gamma =2$, as used for the reference model to maintain comparability of the flexibility parameterization. These assumptions may be relaxed by a parameterized family of kernels and an inference of their parameterization together with the KL parameters^48^. The reconstruction FE model exhibits 50 quadratic elements leading to a spatial evaluation of the flexibility at 101 nodes. This coarser discretization in comparison with the reference model is once again chosen to avoid an inverse crime^49^.

To reduce the random dimensionality, we discretize the unknown random field with the KL expansion from Eq. (2) truncated to $s=6$ terms. Assuming a constant mean, this yields $s+1$ unknown random variables that make up the discrete vector of unknowns $\varvec{\theta }$, namely the mean and the *s* KL parameters. Following Huang et al.^45^, this configuration accounts for $\alpha =98\%$ of the variance of the random flexibility.

By using the KL expansion, we essentially apply a Gaussian process prior on the flexibility. Within this prior probability, the flexibility mean is distributed according to

12 $$\begin{aligned} \mu _{C}\sim \mathscr {N}\left( \mu =5\times 10^{-12}\ \frac{m^2}{N},\ \sigma ^2=\left( 1\times 10^{-12}\ \frac{m^2}{N}\right) ^2\right) \end{aligned}$$

and the KL parameters are endowed with a normal prior:

13 $$\begin{aligned} \xi _{i-1} \sim \mathscr {N}\left( \mu =0\; ,\ \sigma ^2 = \left( 1\times 10^{-11}\frac{m^2}{N}\right) ^2\right) \quad \quad \forall i > 1. \end{aligned}$$

These prior distributions may be interpreted analogously to regularization in optimization. The chosen normal prior on the flexibility mean represents a weak assumption, while the prior on the KL coefficients encodes an assumption on the flexibility variance.

The real noise standard deviations present the ideal choice for the likelihoods standard deviations, because inaccurate measurements are not erroneously interpreted as accurate, and conversely, more accurate measurements are not assumed as excessively noisy, thus leading to a loss of information. In practice, the error or noise characteristics are unknown, but may be estimated from the statistical information gained from repeated measurements. We define the likelihoods with a higher standard deviation than that of the synthetic measurement noise used and thus underestimate the measurements’ accuracy. The numerical values are compiled together with all parameters that are necessary for reproducing the results in the Online appendix S1. The likelihood function for vector-valued measurements in Eq. (10) implies that each eigenfrequency is measured only once and not repeatedly.

**Figure 3:**
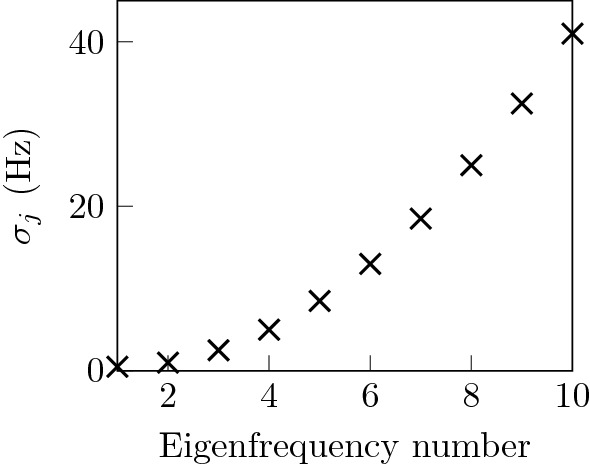
The measurement likelihood standard deviation is expressed as a function of the frequency. The graph shows the chosen quadratic increase of the measurement likelihood standard deviation $\sigma _j$ over the number of the corresponding eigenfrequency. This weighting emphasizes the influence of the first few eigenfrequencies. The higher likehihood standard deviation for the higher eigenfrequencies reflects the expectation that measurement accuracy deteriorates with increasing frequency.

The likelihood’s standard deviation increases quadratically with the number of the corresponding eigenfrequency, see Fig. 3. Matching low eigenfrequencies is thus given more importance.

The slice sampling algorithm generates samples $\varvec{\theta }^{(i)}$ from the posterior in Eq. (3). Multiple chains with different initial values help attenuate the influence of the initial value of the sampled Markov chain alongside the exclusion of burn-in samples from the number of samples used *U*. Evaluating the applied KL expansion at the posterior samples then produces the corresponding samples of the posterior random field.

**Figure 4:**
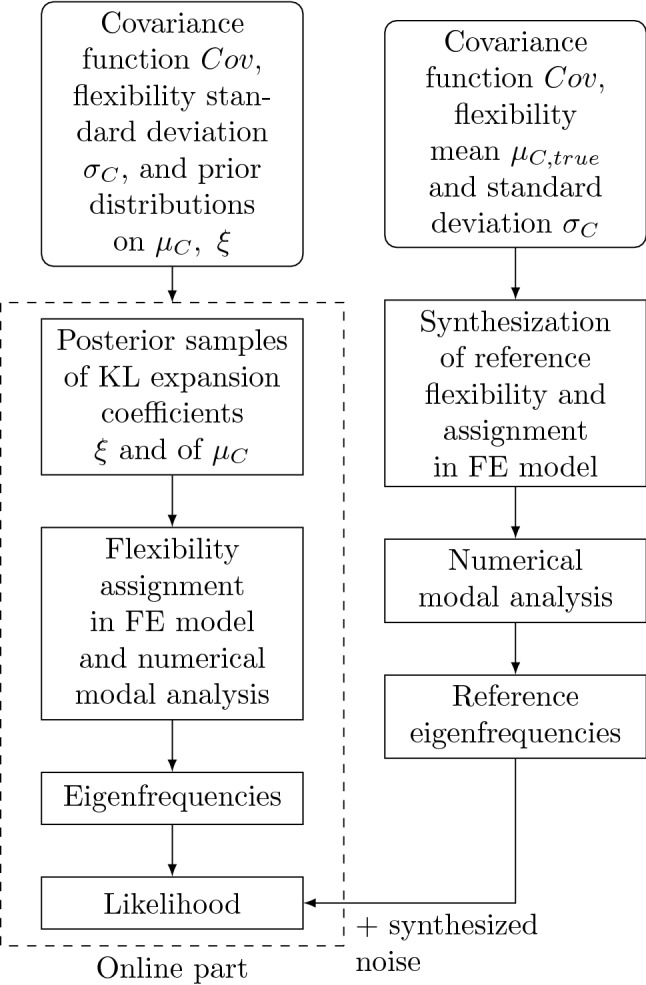
General procedure for reconstructing the reference random field given noisy eigenfrequencies and assuming the reference covariance, priors, and measurement noise characteristics with Bayesian inference. The top part refers to the calculation of the reference eigenfrequency from the reference flexibility. Given noisy observations of these reference frequencies, the aim of the procedure detailed at the bottom is to estimate the reference flexibility. Here, the dashed line marks the part of the inference that must be computed at every step in the chain.

Along with the flexibility’s expected value,

14 $$\begin{aligned} \mu _{C, post}(t_j) = \frac{1}{U}\sum _{u=1}^{U} C^{(u)}(t_j), \end{aligned}$$

we compute confidence intervals that contain 95% of the values of $C^{(u)}(t_j)$ for each position $t_j$. Finally, the root mean square percentage error (RMSPE) with respect to the reference flexibility is obtained as

15 $$\begin{aligned} \varepsilon _{RMSPE} = \sqrt{\frac{1}{k} \sum _{j=1}^{k} \left( \frac{\mu _{C, post}(t_j)-\varvec{C}_{true}(t_j)}{\varvec{C}_{true}(t_j)}\right) ^2} \cdot 100\%. \end{aligned}$$

#### Flexibility identification using deflection measurements from static analysis

The identification of the structural flexibility using static deflection data follows the same general procedure as described in “Flexibility identification using eigenfrequency measurements from modal analysis”. This section does not repeat the steps shared between the two procedures, it highlights the differences instead.

Here, noisy measurements of the simulated static deflections of the cantilever beam with the reference flexibility constitute the data. With these 10 equispaced static deflections, we estimate the unknown reference flexibility $\varvec{C}_{true}$.

Replacing modal with static analysis and eigenfrequencies with static deflections, respectively, in the procedure diagram, see Fig. 4, yields the inversion procedure using static analysis.

Contrary to inversion via modal analysis, we choose a constant likelihood standard deviation for the static analysis. The likelihood follows Eq. (10), where the static deflections are measured once at each equispaced position.

## Results and discussion

This section presents the findings of the present study. “Modal analysis” and “Static analysis” consider the confidence interval of the solution over the beam coordinate and “The effects of signal-to-noise ratio and flexibility correlation length” explores the effects of signal-to-noise ratio as well as flexibility correlation length.

**Figure 5:**
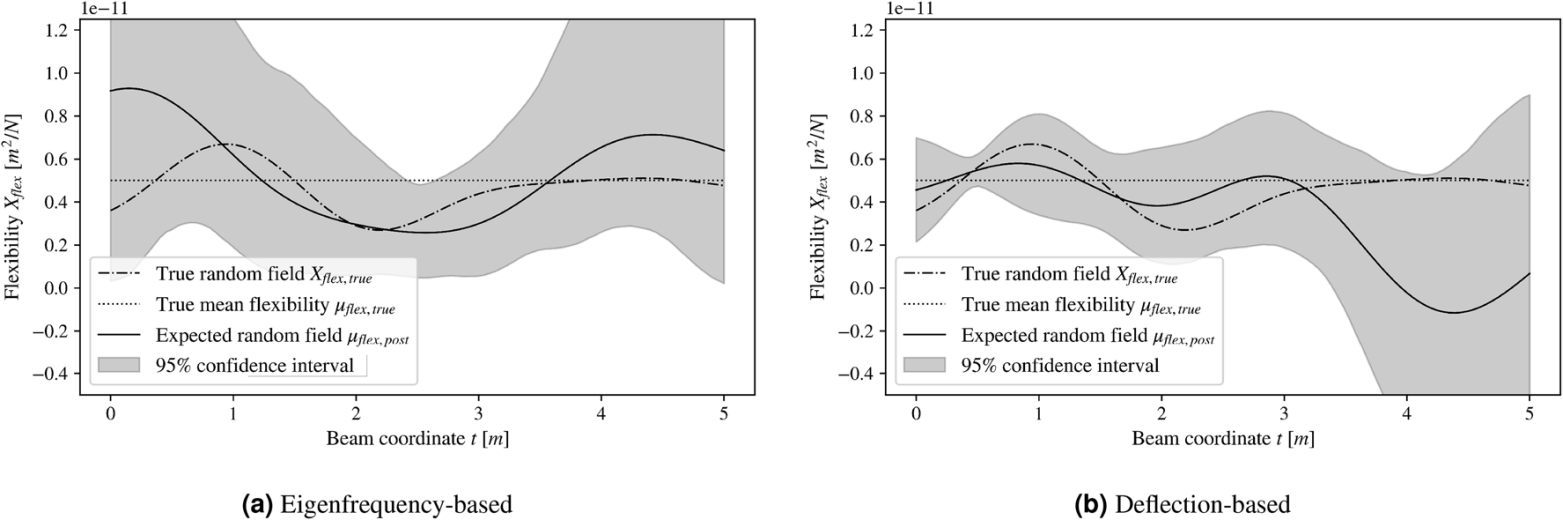
The figures show the results for the inference workflow for a specific reference flexibility. The left graph corresponds to the modal analysis, while the right figure is connected to the static analysis. The respective dashed-dotted lines show the reference flexibility, while the respective solid lines represent its estimated posterior mean. Low heights of the confidence intervals indicate a higher certainty of the inference results at the respective location.

Figure 5 shows the results of the procedure for one exemplary realization of the random flexibility. Here, the dashed-dotted lines mark the a priori unknown reference flexibility. Figure 5a shows the result using the dynamic method and Fig. 5b illustrates the result for the static deflection-based method for comparison. Note that the proposed Bayesian approach yields a chain of samples for $\theta _i$. These samples can be used to estimate the posterior distribution’s higher statistical moments in addition to mean and variance. Restricting the analysis of the results to mean and variance would disregard any skewness of the posterior at any location, which is visible in Fig. 5 through the asymmetrical confidence intervals. Additionally, note that the procedure has produced a non-stationary posterior random field as these moments are not constant over the beam length.

The following paragraphs interpret the confidence interval properties along the beam coordinate *t* based on a total of 100 realizations of the flexibility such that the interpretations are applicable in a general sense.

### Modal analysis

With the eigenfrequency-based approach and with the chosen likelihood structure, the size of the confidence interval is roughly constant along the beam coordinate *t*. The present choice of the first 10 eigenfrequencies thus leads to a comparable amount of flexibility information for all spatial positions.

Avoiding non-physical signs of the flexibility is straightforward using the eigenfrequency-based model, since negative flexibility leads to a negative squared eigenfrequency. For this case, the likelihood of corresponding solution candidates is simply set to zero and we thus obtain a purely positive estimation of the flexibility here.

### Static analysis

With the static deflection-based approach, the confidence interval increases as the distance from the clamping grows. This is consistent with the intuition that the bending moment within the beam varies linearly along the beam axis, with the maximum absolute value being at the clamping. Because the impact of flexibility fluctuations on the deflection depends directly on the bending moment, these fluctuations have their biggest impact close to the clamped boundary. Conversely, the deflections contain proportionately more information about the flexibility on the left side than on the right side. This facilitates error propagation from the left to the right part of the domain and it finally leads to the narrow confidence interval in the left part and the wide confidence interval in the right part of the beam.

With the static deflection-based model, some issues may arise with the flexibility’s sign, owing to the Gaussian random field’s support $C(t_j)\in \mathbb {R}$ within the reconstruction. Here, the estimation violates the physical restriction of the flexibility being positive at some locations on the right side of the beam. The reason for this is a mixture of the characteristics of the beam and the assumed measurement noise. The cantilever beam exhibits a small bending moment on its right side, leading to a small curvature on this side. To simulate the deflection measurements, we add synthetic Gaussian noise to the deflections. In regions on the right side with a low reference curvature, the curvature of the noise is likely to dominate the total curvature within the simulated measurements. As the bending moment links the flexibility and the curvature, the reconstruction essentially estimates the curvature of the beam. This explains why the curvature component resulting from the synthetic measurement noise may propagate to the estimated flexibility and consequently lead to negative values for the flexibility in some cases.

### The effects of signal-to-noise ratio and flexibility correlation length

This study focuses on investigating and comparing two non-destructive methods for material parameter identification. To study the efficacy of the dynamic and static method, we demonstrate the strategic variation of the inverse problem’s configuration. Specifically, we expect both larger correlation lengths of the flexibility and larger signal-to-noise ratios to improve the inversion quality and did indeed obtain these expected results.

**Figure 6:**
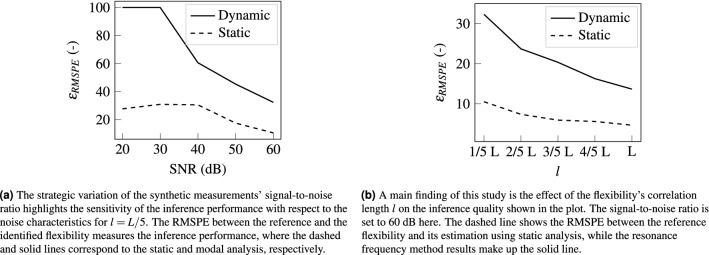
Comparison of the methods’ performance influenced by changing inverse problem configurations. The left graph shows the effect of changing signal-to-noise ratios, while the right graph shows the impact of flexibility correlation length.

The effect of the signal-to-noise ratio (SNR) on the solution quality is investigated with a systematic variation of noise standard deviation, see Fig. 6a. To obtain representative results, the described procedure is carried out for 100 unique realizations of the reference flexibility per signal-to-noise ratio. The error described in Eq. (15) is then averaged over the 100 realizations. The error decreases non-lineary for the chosen SNR scale. Comparatively low signal-to-noise ratios produce a plateau in the error. After a kink in the curve, higher measurement noise entails flattening error behavior. We observe a consistently lower RMSPE when employing the approach using static deflection measurements and a higher order of error convergence for the resonance frequency method. Note that more accurate measurements can be obtained in practice by averaging over several repeated measurement runs.

The variation of the flexibility correlation length shown in Fig. 6b exhibits the expected outcome. The error decreases non-linearly with increasing ground truth correlation lengths. The error gap between the static and dynamic methods narrows with growing correlation lengths. The comparatively large errors in the small correlation length regime result from the higher complexity of the unknown function. This in turn corresponds to an increasingly complex parameter space that the inference procedure needs to traverse. On the contrary, an infinitely large correlation length would correspond to a constant flexibility. This represents the simplest case and we expect the smallest errors here.

Concerning the static analysis, this study does not account for uncertainty in the load and its application to the specimen. These uncertainties propagate through the system to the deflections. Additionally, the measurement of the deflections is subject to measurement errors. Measurement noise challenges for micro-scale applications are linked to physical restrictions in optics^50^. Macro-scale applications like the one studied in this paper on the one hand rely on methods such as digital image correlation^51^. On the other hand, they use optical active or passive marker systems that typically involve camera setups^52^. Here, a compromise must be found between the covered area and the camera distance, the two of which are coupled by the viewing angle. Maletsky et al.^53^ report a non-linear relationship between camera distance and SNR and find an overall SNR of 45 dB for a generic setup. In fact, SNRs of higher than 60 dB are already achievable for dynamic response measurement setups^54^. Accounting for this measurement accuracy of dynamic methods exceeding that of static methods^8^, an unfavorable light is cast on the modal analysis.

This study considers the modal and static analyses of an identically configured, clamped cantilever beam and does not account for uncertainty in the boundary conditions. However, an experimental modal analysis is typically conducted with free-free boundary conditions that are more accurately reproducible in practice than other mounting conditions^55^. Here, this benefit of the method is traded for comparability with respect to the static analysis.

Debruyne et al.^39^ find the usefulness of experimental modal analysis doubtful for their model updating procedure, when the measurement quality is not excellent. Their conclusion is confirmed by our results that stem from a setting with deterministically known modeling errors. Mehrez et al.^38^ state that their number of data points prove suitable for their problem configuration. Our results complement this by setting the SNR and error into relationship, which enables an estimate for the required number of data points to achieve an error tolerance given the SNR of a single measurement. Their confidence region makes up for $\approx \!30 \%$ of the mean value. Our resonance frequency method matches this estimation accuracy for high signal-to-noise ratios and ground truth random field correlation lengths close to or greater than *L*. This is due to the gradient-agnostic sampling algorithm used in this study on the one hand and due to the difference in information provided to the method on the other hand, as local instead of global data is used in the study Mehrez et al.^38^.

## Conclusion

We develop a new Bayesian resonance frequency method with reduced stochastic dimensionality for identifying the spatially varying structural flexibility of a cantilever beam. It exhibits a major advantage compared to existing non-destructive methods for determining local macro-scale material properties using dynamic data. As it does not rely on local information as conventional methods do, it can operate without line-of-sight to the specimen. This is especially valuable in the context of the advent of functionally graded materials. The latter is furthering spatially varying material properties within geometrically complex assemblies. Here, our method enables non-destructive testing when undercuts are present.

We obtain results for the non-linear error characteristics with respect to SNR and the flexibility correlation length. Considering the influence of SNR highlights that a saturation of the error occurs at low signal-to-noise ratios. These results are set in relation to those obtained from applying the Bayesian procedure to the cantilever subjected to static linear elastic loading.

In conclusion, using identical noise and flexibility correlation length characteristics:

- inversion based on static deflections yields lower absolute errors.
- the confidence interval widens with growing distance from the clamping for the static approach.
- the confidence interval height using the dynamic approach stays constant along the beam.

We further conclude that, generally:

- larger flexibility correlation lengths lead to improved reconstruction.
- higher signal-to-noise ratios reduce the estimation error.

In practice, the choice of method should carefully consider the reproducibility of the real boundary conditions within the numerical models and especially the signal-to-noise ratios achievable by the experimental setups.

Currently, no reliable data describing the spatial randomness of material properties are available, and Matérn covariance models or special cases like isotropic exponential kernels are used as a fallback, see^48^. Identifying the covariance from such data systematically for common material classes, the connected manufacturing processes, and engineering applications that introduce heterogeneity would eliminate the need for many assumptions that are currently necessary. Future research needs to study the influence of these identified covariance models and their respective parameters on the efficacy of our method. This may include the construction of compound covariance kernels from base kernels, for example using addition or multiplication, see Hofmann et al.^56^. This property could be used to combine kernels across spatial dimensions and model, among others, anisotropically heterogeneous materials.

This paper shows the solution of the inverse problem for a single quantity of interest that depends on a spatial coordinate. In practice, more than one parameter can be relevant. In the context of isotropic materials, the shear modulus or Poisson’s ratio as well as the mass density may be relevant. For anisotropic materials, the spatial components of the elastic properties are additionally needed to fully characterize the material. This complicates the inverse problem. However, taking into account for additional information promises to mitigate these effects. For some material classes, the spatial components of the elastic properties are linearly correlated. Specifically for wood, the Young’s modulus in a tree’s growth direction correlates linearly with the Young’s modulus in the radial direction orthogonal to the growth rings. Often, Pearson’s coefficient for linear correlation exceeds $r=0.5$ here. Preliminary investigations have shown that incorporating knowledge of the cross-correlation is not uniformly beneficial. Conversely, the method’s success depends on the cross-correlation amplitude and the algorithm used to sample from the posterior distribution, among others. Future research needs to address this research gap and produce encompassing results that serve as a guideline for researchers.

## Supplementary Information

Supplementary Information.

## Data Availability

The raw data generated during the current study are available from the corresponding author on reasonable request.
