# Supplementary material for: Identification of a cantilever beam’s spatially uncertain stiffness
Source: Sci Rep. 2023 Jan 20;13:1169. doi: 10.1038/s41598-023-27755-5 (PMC9860023; doi:10.1038/s41598-023-27755-5)
Supplement: Supplementary file 1 — Supplementary Information. [file 41598_2023_27755_MOESM1_ESM.pdf]

Identification of a Cantilever Beam's Spatially Uncertain Stiffness

Karl-Alexander Hoppe, Martin G. T. Kronthaler, Kian Sepahvand and Steffen Marburg

A Static Analysis - Model Description

Consider a cantilever beam subjected to static loading F with the deflection response $w(t)$ at a specific position shown in Figure 7. This results in a linear

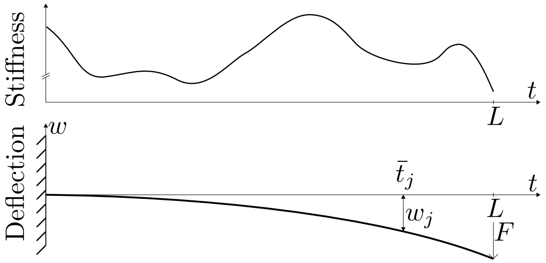

Fig. 7: The top graph shows an arbitrary exemplary stiffness curve of a cantilever beam and the lower graph shows the beam's static deflection when subjected to a load.

forward operator with analytical solutions, see [40].

B Parameters for the Example Calculations

Table 1: Beam properties

Length of beam L	5 m
Height h	0.1 m
Width g	0.1 m

Table 2: Reference random field properties

Mean Young's modulus $\mu_{E,true}$	2×10^{11} N/m^2
Mean material flexibility $\mu_{C,true}$	5×10^{-12} m^2/N
Dimensionality of discretized random field	100
Standard deviation to mean ratio (flexibility based) $\sigma_C/\mu_{C,true}$	0.2
Standard deviation for flexibility field σ_C	1×10^{-12} m^2/N
Correlation kernel type	exponential

Table 3: Random field properties used for inversion

Number of KL Parameters s	6
Correlation kernel type	exponential
Minimum covered variance α for $l = 1$	98.13%

Table 4: Bayesian inversion parameters

Initial values	$\mu_C^{(0)} \sim \mathcal{N}(5 \times 10^{-12} \frac{m^2}{N},$ $(1 \times 10^{-14} \frac{m^2}{N})^2)$ $\xi_{d,i}^{(0)} \sim \mathcal{N}(0\ \frac{m^2}{N},$ $(1 \times 10^{-15} \frac{m^2}{N})^2) \quad \forall i$

B.1 Static Analysis

Table 5: General parameters

Discretization ground truth	400 integration points
Discretization reconstruction	115 integration points
Force	3,300 N
Maximal absolute deflection	7.21×10^{-2} m

Table 6: Bayesian inversion parameters

Number of chains	8
Total number of samples per chain	7,000
Number of burn-in samples	2,000
Total number of used samples U	40,000
Priors	$\mu_{prior,\mu} = 5 \times 10^{-12}\ \frac{m^2}{N}$<br>$\sigma_{prior,\mu} = 5 \times 10^{-13}\ \frac{m^2}{N}$<br>$\sigma_{prior,\xi_{i-1}} = 5 \times 10^{-12}\ \frac{m^2}{N}\ \forall i > 1$

B.2 Modal Analysis

Table 7: General parameters

Discretization ground true	100 quadratic elements<br>201 degrees of freedom for deflection
Discretization reconstruction	50 quadratic elements<br>101 degrees of freedom for deflection
Number of computed eigenvalues	10
Lowest computed eigenfrequency	3.625 Hz
Highest computed eigenfrequency	863.8 Hz

Table 8: Bayesian inversion parameters

Number of chains	4
Total number of samples per chain	3,500
Number of burn-in samples	1,000
Total number of used samples U	10,000
Priors	$\mu_{prior,\mu} = 5 \times 10^{-12}\ \frac{m^2}{N}$<br>$\sigma_{prior,\mu} = 1 \times 10^{-12}\ \frac{m^2}{N}$<br>$\sigma_{prior,\xi_{i-1}} = 1 \times 10^{-11}\ \frac{m^2}{N}\ \forall i > 1$

C Methods for Second Order Random Fields

C.1 Multivariate Normal Distribution

The normal distribution can be generalized for random vectors x, leading to the multivariate Gaussian (or normal) distribution. Its probability density is defined as [20]:

$$f(\boldsymbol{x}) = \frac{1}{(2\pi)^{n/2} \cdot |\boldsymbol{\Gamma}|^{1/2}} \cdot \exp\left(-\frac{1}{2}(\boldsymbol{x}-\boldsymbol{\mu})^T \boldsymbol{\Gamma}^{-1}(\boldsymbol{x}-\boldsymbol{\mu})\right), \tag{16}$$

with $\boldsymbol{\mu}$ being the mean vector and $|\boldsymbol{\Gamma}|$ being the determinant of the covariance matrix $\boldsymbol{\Gamma}$.

C.2 Properties of the Covariance Function

The properties of the covariance functions considered here follow Abrahamsen [17]: First,

$$Cov(t,t) = 1, \tag{17}$$

and it follows that

$$|Cov| \leq 1. \tag{18}$$

Second, it must be symmetric, meaning

$$Cov(t,t') = Cov(t',t) \quad \forall t,\ t', \tag{19}$$

and finally, the covariance must be positive semi-definite, meaning

$$\sum_{i=1}^{k}\sum_{j=1}^{k} c_i\, c_j\, Cov(t_i,t_j) \geq 0\ \forall\ k,\ \{t_1,...,t_k\},\{c_1,...,c_k\}. \tag{20}$$

C.3 Numerical Treatment of the KL Expansion

The eigenvalues λ_i and the eigenfunctions $\varphi_i(t)$ from (2) are the solutions to a Fredholm integral equation of the second kind

$$\int_B Cov(t,\ t')\ \varphi_i(t')\ dt' = \lambda_i\ \varphi_i(t). \tag{21}$$

The eigenvalues λ_i and eigenfunctions φ_i are ordered as $\lambda_1 \geq \lambda_2 \geq \lambda_3 \cdots$. For a Gaussian random field, the KL expansion leads to a representation of the field with uncorrelated ξ_i. Betz et al. [42] give an overview of the feasible methods for solving the integral eigenvalue problem numerically. Here, we use the Nyström method, where the integral in (21) is approximated by a numerical

integration scheme. This leads to

$$\sum_{j=1}^{k} v_j \; Cov(t,\ t_j) \; \varphi_i(t_j) \; dt_2 = \lambda_i \; \varphi_i(t), \tag{22}$$

with the integration weights $v_j,\ j \in \{1, 2, \ldots, k\}$. We use a piece-wise constant integration scheme with integration weights given as the length between the surrounding grid points

$$v_j = \begin{cases} \frac{t_2 - t_1}{2} & \text{if } j = 1 \\ \frac{t_k - t_{k-1}}{2} & \text{if } j = k \\ \frac{t_{j+1} - t_{j-1}}{2} & \text{else.} \end{cases}$$

This leads to the matrix eigenvalue problem

$$\boldsymbol{C}\boldsymbol{V}\boldsymbol{y}_i = \lambda_i \boldsymbol{y}_i, \tag{23}$$

with the covariance matrix

$$\boldsymbol{C} = C_{ij} = Cov(t_i,\ t_j), \tag{24}$$

and the matrix of integration weights

$$\boldsymbol{V} = diag(v_j), \tag{25}$$

see [42]. The eigenvectors $\boldsymbol{y}_i = y_{ji} \approx \varphi_i(t_j\ )$ in Equation (23) approximate the eigenfunctions φ_i. For equidistant grid points t_i and neglecting boundary effects, a scaled identity matrix approximates the matrix of integration weights. The eigenvalues of the covariance matrix need to be scaled with the distance between the grid points for consistency with Equation (23). To consistently account for the random field variance as per Mercer's theorem, the eigenvectors $\widehat{\boldsymbol{y}}_i$ are normalized as

$$\boldsymbol{y}_i = \frac{\widehat{\boldsymbol{y}}_i}{l_i} \quad \text{with} \quad l_i = \sqrt{\sum_{j=1}^{k} v_j (y_{ji})^2}, \tag{26}$$

see [42]. The direction of the eigenvectors $\boldsymbol{y}_i$ needs to be fixed in order for the KL expansion to be a unique representation of a random field in the context of the inverse problem. We prescribe a positive first element of each eigenvector

$$y_{i1} \overset{!}{>} 0 \quad \forall i \tag{27}$$

to fix the direction.
